# Application of Multi-Channel Synchronized Dynamic Strain Gauges in Monitoring the Neutral Axis Position and Prestress Loss of Box Girder Bridges

**DOI:** 10.3390/s24113489

**Published:** 2024-05-28

**Authors:** Shu-Ken Lin, Yi-Ching Lin, Jian-Hua Tong, Han-Ting Cheng, Hsin-Chu Tsai, Jui-Lin Wang

**Affiliations:** 1 Department of Civil Engineering, National Chung Hsing University, 145 Xingda Road, South District, Taichung City 402, Taiwan; sklin@nchu.edu.tw; 2 Department of Intelligent Technology and Application, Hungkuang University, No. 1018, Section 6, Taiwan Boulevard, Shalu District, Taichung City 433, Taiwan; jhtong@sunrise.hk.edu.tw; 3 Department of Public Works, Taiwan Water Corporation, No.2-1, Section 2, Shuangshih Road, North District, Taichung City 404, Taiwan; sinsintina@yahoo.com.tw; 4 China Engineering Consultants, Inc., Floor 28, No.185, Section 2, Sinhai Road, Da-an District, Taipei City 106, Taiwan; hsinchu@ceci.org.tw (H.-C.T.); wwwrainy2001@gmail.com (J.-L.W.)

**Keywords:** multi-channel dynamic strains, edge computing, N.A. position, prestressed concrete box girders, structural health monitoring

## Abstract

The aim of this paper was to explore the application of multi-channel synchronized dynamic strain gauges in monitoring the neutral axis (N.A.) position of prestressed concrete box girders. The N.A. position has recently been proposed as an indicator for monitoring the health of bridge structures. Laboratory experiments were conducted on a prestressed T-beam under different prestress level conditions to investigate the correlation between the prestress magnitude and the N.A. position. In the development of the multi-channel synchronized dynamic strain gauges, edge computing was employed to significantly reduce the amount of data transmitted from the sensor nodes on-site. In edge computing, only the dynamic strain response caused by the maximum vehicle load in each minute is transmitted. This approach greatly enhances the monitoring efficiency and enables the realization of on-site non-computer-based monitoring systems. The laboratory test results of the prestressed T-beam showed that the N.A. position tends to move slightly downward as the prestress force increases. In other words, when the prestress force decreases due to loss, the N.A. position exhibits a slight upward movement. This study selected a newly constructed prestressed box girder as the subject for on-site measurement of the N.A. position using multi-channel synchronized dynamic strain gauges shortly after the prestress was applied. The on-site monitoring data indeed revealed a gradual upward movement of the N.A. position. This phenomenon confirmed that soon after the completion of prestressed concrete bridges, there is a gradual loss of prestress due to the significant shrinkage and creep effects of the early-age concrete. The on-site monitoring result aligned with the findings from the laboratory experiments, where the N.A. position was observed to move upward as the prestress decreased.

## 1. Introduction

When vehicles or trains pass over a bridge, the main girder directly bears the live loads. Due to prolonged traffic loading and natural environmental erosion, the main girder is inevitably subject to structural deterioration and damage. To ensure the safety of the bridge, it is necessary to develop structural health-monitoring methods for the main girder. Currently, the commonly used indicators for monitoring the structural health of the main girder, such as the vibration frequency, static displacement, and static strain, are significantly affected by changes in the environmental temperature, making it difficult to use as quantitative indicators of a bridge’s structural health [1,2,3,4,5,6,7,8].

Since the time taken for vehicles to pass over a bridge is very short, typically only a few seconds, the dynamic strain induced on the main girder during this short time period is unlikely to be affected by temperature variations. Therefore, the strain variation caused by vehicle loading can be considered a pure mechanical strain. This vehicle-induced mechanical strain is closely related to the stiffness of the main girder. Consequently, in recent years, there has been considerable research on using dynamic strain as a structural health-monitoring indicator [9,10,11]. Additionally, the N.A. position of the main girder is related to the centroid of the cross-sectional stiffness. It is highly sensitive to changes in the cross-sectional area and stiffness of the girder. Therefore, the N.A. position is also sensitive to local damage to the main girder, and there has been extensive research on this topic as well [12,13,14,15,16,17,18]. Currently, most of the literature on measuring the N.A. position primarily focuses on bridge damage detection, with relatively fewer studies exploring the influence of prestress variations on the N.A. position. However, prestress loss is a major safety concern for prestressed bridge structures, especially the loss caused by the corrosion of prestressed steel tendons. There is a need to explore the possible application of monitoring the N.A. position to detect prestress loss.

The N.A. position refers to the location within the cross-section of a beam where the normal stress/strain is zero under a load. For concrete beams, if no prestress (axial force) is applied, the N.A. will pass through the centroid of the cross-section. However, the presence of prestress (axial force) or damage will cause the N.A. position to deviate from the centroid. Therefore, the N.A. position can potentially be used for monitoring the prestress condition of concrete beams.

To measure the N.A. position on a cross-section, it is necessary to install at least two strain gauges and then utilize the fundamental assumption of the linear distribution of the bending strain in the beam section to infer the location where the strain is zero. As vehicles pass over a bridge, they induce dynamic strain responses. Typically, sampling frequencies can reach over 40 Hz (40 data points per second), allowing for complete and accurate recording of the dynamic strain responses caused by passing vehicles, which can be used for subsequent analysis of the N.A. position in the cross-section. Continuous monitoring requires dealing with the challenge of large volumes of data output from multi-channel dynamic strain gauges. Therefore, most monitoring sites currently employ computer-based equipment with operating systems to capture, transmit, and store the vast amount of data. However, if the computer’s heat dissipation is not properly managed, it can lead to system crashes. Additionally, analyzing the huge outputted data is also a practical challenge.

The two main research themes of this study are investigating the influence of the prestress variation on the N.A. position and developing multi-channel synchronized dynamic strain gauges with edge computing. Regarding the effect of the prestress variation on the N.A. position, laboratory tests on a prestressed concrete beam were first conducted. Prestressed steel tendons were anchored on hollow hydraulic jacks, and the prestress magnitude was controlled by adjusting the extension or shortening of the tendons through changes in the hydraulic jack’s stroke. Flexural tests of the beam were then performed under specific prestress levels to determine the N.A. position and analyze the influence of the prestress variation.

Since prestressed concrete beams experience prestress loss during early age due to concrete shrinkage and creep, the field experiments in this study focused on newly constructed box girders to observe how the N.A. position is affected by prestress loss. To enhance the efficiency of the field monitoring system, edge-computing technology was utilized to develop multi-channel synchronized dynamic strain gauges, which transmit only the key data every minute. These key data represent the maximum dynamic strain induced by the heaviest vehicle within that minute. This approach significantly enhances the monitoring efficiency, allowing for the replacement of traditional computer-based monitoring systems with microcontroller systems. This meets the requirements for low power consumption and long-term stability in the field.

This paper begins by presenting the laboratory test results of a prestressed T-beam. Subsequently, it discusses the development of multi-channel synchronous dynamic strain gauges using edge computing. The final part of the paper introduces the practical application of the multi-channel synchronized dynamic strain gauges developed in this study on a newly built box girder bridge on site.

## 2. Laboratory Test

### 2.1. Description of the Specimen

**Figure 1:**
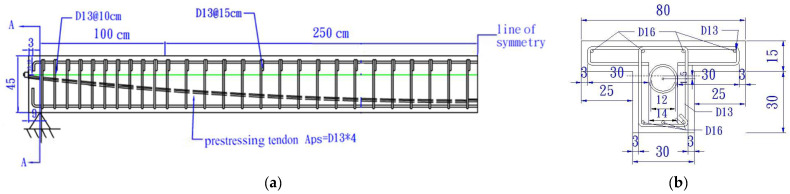
Prestressed T-beam reinforcement and prestressing tendon configuration: (**a**) side view; and (**b**) cross-section view.

A prestressed T-beam with a length of 7 m was fabricated as a specimen to investigate the correlation between the N.A. position and the prestress variation. The materials used for specimen fabrication included concrete with a compressive strength of 420 kgf/cm^2^ and steel reinforcement with a yielding strength of 4200 kgf/cm^2^. Figure 1a shows a side view of the steel reinforcement and prestressing tendon configuration on the left half of the symmetrical axis of the T-beam specimen, while Figure 1b illustrates the cross-section at the left end point. The nominal diameters of the main reinforcement and stirrups are 16 mm and 13 mm, respectively, and four high-tensile steel tendons with a diameter of 13 mm were used for applying prestress to the specimen.

**Figure 2:**
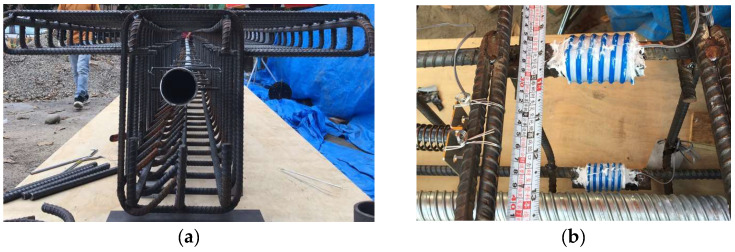
T-beam specimen: (**a**) assembly of the reinforcement; and (**b**) waterproof strain gauges.

Figure 2a depicts a photo of the completed assembly of the reinforcement for the T-beam specimen. In preparation for subsequent tests to measure the strains caused by loading for calculating the N.A. position, strain gauges were attached to the upper and lower layers of the reinforcement at the midpoint cross-section of the T-beam, as shown in Figure 2b, with waterproof protection.

**Figure 3:**
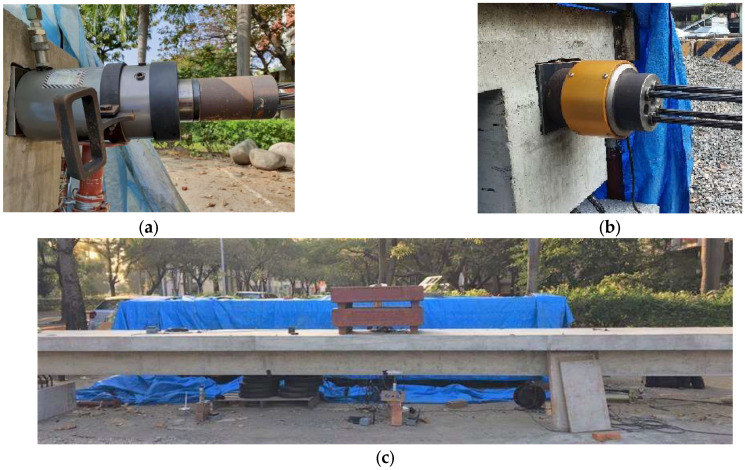
Prestress-adjusting device and measurement: (**a**) hollow hydraulic jack; (**b**) load cell; and (**c**) loading test.

During the experiment to induce prestress variation, the tendons were anchored on hollow hydraulic jacks and load cells at the two ends of the specimen, as depicted in Figure 3a,b respectively. Adjusting the stroke of the hollow hydraulic jacks allows for the tightening or loosening of the tendons, thereby achieving the function of adjusting the prestress magnitude. The change in the prestress was measured by the load cells. To obtain the flexural deformation of the T-beam and determine the position of the N.A. under different prestress levels, loading tests were conducted by applying weights at the midpoint of the beam, as illustrated in Figure 3c.

### 2.2. Effect of Prestress on N.A. Position

**Figure 4:**
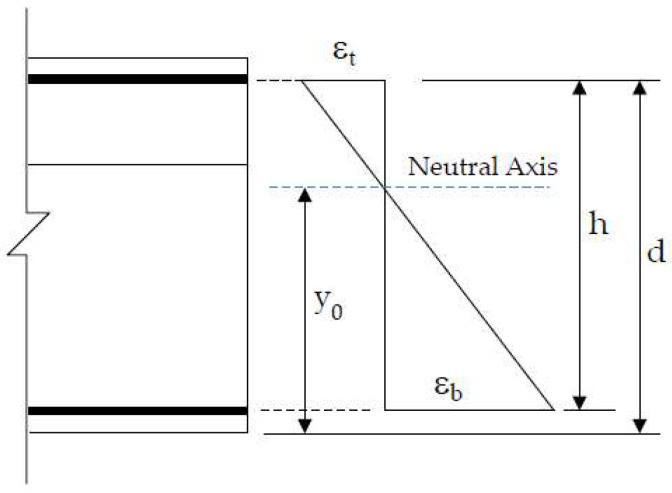
Schematic diagram of the strain distribution and NA position.

Firstly, a loading test was conducted on the T-beam without applying prestress, using two 500 kgf weights for the loading and unloading experiments. During the loading test, strain gauges attached to the top and bottom layers of the reinforcement were used to measure the strains. Under the action of external loading, the strain responses recorded by the two strain gauges allow for the calculation of the N.A. position based on the assumption of the plane section remaining plane during beam flexure, as illustrated in Figure 4 and described by the following equation:

(1) $$y_{0}=\frac{\varepsilon_{b}h}{\varepsilon_{b}-\varepsilon_{t}}+d-h$$

where

$y_{0}$: the distance from the bottom of the beam to the N.A.
$\varepsilon_{t}-\varepsilon_{b}$: the strains of the top and bottom layers of the reinforcement, respectively.
h: the distance between the two strain gauges.
d: the distance from the strain gauge on the top layer of the reinforcement to the bottom of the beam.

During the fabrication process of the specimen, measurements were taken in advance to determine that the distances from the top layer and bottom layer of the reinforcement to the bottom of the beam were 39.94 cm and 5.07 cm, respectively. Therefore, in Equation (1), we already know d = 39.94 cm and h = 34.87 cm. Hence, during the loading test, once the strain responses ($\varepsilon_{t}-\varepsilon_{b}$) were measured, the position of the N.A. can be calculated using Equation (1).

**Figure 5:**
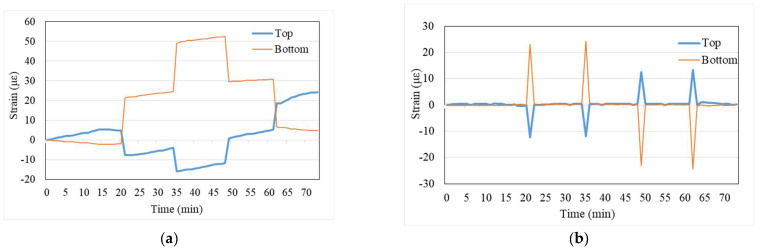
T-beam load test without prestressing: (**a**) measured strain responses; and (**b**) mechanical strains.

Figure 5a shows the time–history variations of the strains measured by the strain gauges at the mid-span cross-section of the test beam. It can be observed that during loading, the bottom of the beam experiences tensile strains (orange line), while the top undergoes compressive strains (blue line). Even in the absence of load variations, there is a gradual increase in the strains, attributed to temperature changes during the test period. Taking advantage of the slow nature of the temperature changes, the difference between two data points within a short time span is treated as the amount of mechanical strain without a temperature change. Thus, by subtracting the preceding data from the subsequent data in Figure 5a, Figure 5b is obtained. From Figure 5b, significant mechanical strains induced by the application and removal of loads can be clearly observed, while there are no apparent mechanical strains during periods of no load variation.

Table 1 summarizes the strain responses of the top and bottom steel bars in the T-beam load tests without prestressing. The strains obtained in each load test can be used to calculate the N.A. position via Equation (1). The experimental results show that the N.A. position ranges between 28.03 and 29.04 cm. The last column in the table presents the average N.A. position as 28.39 cm, which is quite consistent with the centroid of the cross-section of the T-beam, 27.86 cm.

Next, load tests were conducted on the T-beam under different prestressing conditions. During the tests, the prestress variation was achieved by adjusting the stroke of a hollow hydraulic jack, while the prestress values were recorded using a load cell at the opposite end. After applying each level of prestress, a load test was performed and the steel bar strain responses were recorded. The load test process involved placing a 500 kgf weight at the midpoint of the beam for a certain period before removing the weight. This procedure was repeated for load tests under different prestress levels.

**Figure 6:**
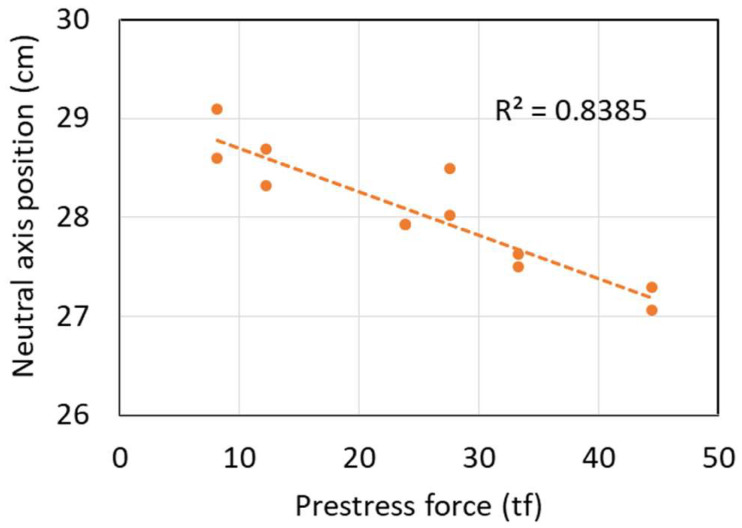
Relationship between the N.A. position and the prestress force.

Table 2 summarizes the results of the load tests on the T-beam under different prestressing conditions. The data from the table are plotted in Figure 6, showing the relationship between the N.A. position and the prestress force. It can be observed that the N.A. position tends to decrease with increasing prestress force. In other words, if prestress loss occurs in the beam, an upward movement of the N.A. position should be observed.

## 3. Edge Computing and Multi-Channel Synchronized Dynamic Strain Gauges

### 3.1. Edge Computing

Installing dynamic strain gauges on bridges allows for real-time monitoring of the vehicle-induced strains when vehicles pass over the bridges. The monitored strains enable the understanding of the mechanical behavior of the bridges and facilitate effective safety management. However, in practice, dynamic strain monitoring requires a considerable amount of data recording and transmission, which can impose a significant burden on the field monitoring systems, potentially leading to issues such as high power consumption and system instability.

In a previous study [19], the authors proposed the use of edge computing to solve the problem of large amounts of data transmission in existing dynamic strain-monitoring systems. The fundamental algorithms of edge computing involve using sliding windows and moving average methods to identify the local maximum and minimum values of the strain data caused by vehicles. Then, the strain caused by vehicles can be obtained by subtracting the local maximum value from the local minimum value. Through the comparative process of the strain induced by each vehicle within a minute, the relative maximum strain per minute can be found, which is referred to as the key data in this study.

**Figure 7:**
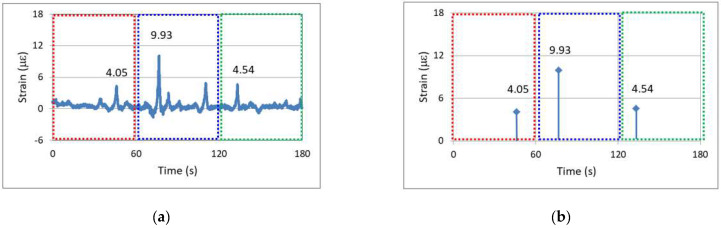
Dynamic strain of the investigated bridge: (**a**) continuous data output; and (**b**) outputting of a maximum value every minute [19].

Figure 7 can be used to illustrate the concept of edge computing’s application in bridge structural health monitoring. In Figure 7a, which depicts the traditional approach with a sampling frequency of 100 Hz, the continuous dynamic strain responses were captured over three minutes. The figure shows short-duration, high-amplitude dynamic strains caused by vehicles passing over the bridge. In Figure 7b, on the other hand, smart sensor nodes with edge computing were deployed on-site. After computation, only the relative maximum dynamic strain responses within each minute were outputted. For example, the values 4.05, 9.93, and 4.54 με shown in Figure 7b represent the relative maximum dynamic strains for the first, second, and third minutes, respectively. These maximum dynamic strain values within each minute are the key data for edge computing. After such processing, the need to record and transmit 18,000 data points over three minutes is significantly reduced to only transmitting three data points. Therefore, the use of edge computing at sensor nodes can greatly enhance the efficiency of the long-term monitoring of bridge dynamic strains.

### 3.2. Multi-Channel Synchronized Dynamic Strain Gauge

**Figure 8:**
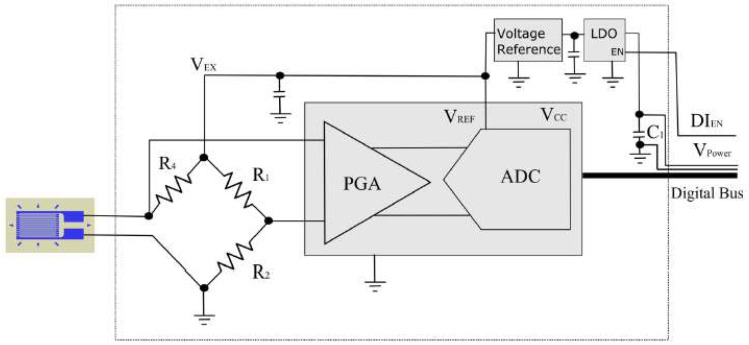
High-precision dynamic strain signal acquisition circuit.

This study adopts resistive strain gauges and utilizes a 1/4 Wheatstone bridge configuration, as illustrated in Figure 8. Figure 8 primarily features an Analog-to-Digital Converter (ADC) with built-in Programmable Gain Amplifiers (PGAs). A serial digital bus is controlled by a microcontroller to adjust the amplification ratios and ADC parameters. This configuration minimizes the analog signal transmission line and effectively reduces the electromagnetic interference and temperature effect on wires. A 24-bit Delta-Sigma-type ADC is chosen for the signal conversion, significantly improving the signal resolution within the required dynamic sampling frequency range while achieving low noise conversion results. The bridge section (R_1_, R_2_, R_4_) incorporates high-precision resistors with ultra-low-temperature coefficients to mitigate the environmental temperature’s impact on the bridge. Additionally, it eliminates any drifts and faults that might arise from the traditional calibration resistors connected in parallel, allowing direct zeroing by firmware.

A sensor node with edge computing requires a processor, a memory for data processing, standard industrial communication interfaces for communication with peripheral devices, a digital I/O for controlling the communication modules, strain acquisition circuits, and a power supply circuit to drive the node’s operation. This study utilizes microcontrollers based on Reduced Instruction Set Computer (RISC) technology as the core of the system development. These chips come with built-in processors, memory, communication, and I/O interfaces.

Through precise timing and timer interrupt firmware design, the system connects multiple sets of high-precision dynamic strain signal acquisition modules via the Serial Peripheral Interface (SPI) interface. It controls the operation of the PGA and ADC and receives digitized data. All the communication between the node and the strain signal acquisition modules is digital, utilizing standard Ethernet connections, ensuring that the strain-acquisition signals are not affected by external electromagnetic interference. For communication with cloud servers, the node connects to Narrowband Internet of Things (NB-IoT) communication modules via the Universal Asynchronous Receiver/Transmitter (UART) interface. The microcontroller sends AT commands for connection control, enabling the node to directly connect to cloud servers.

**Figure 9:**
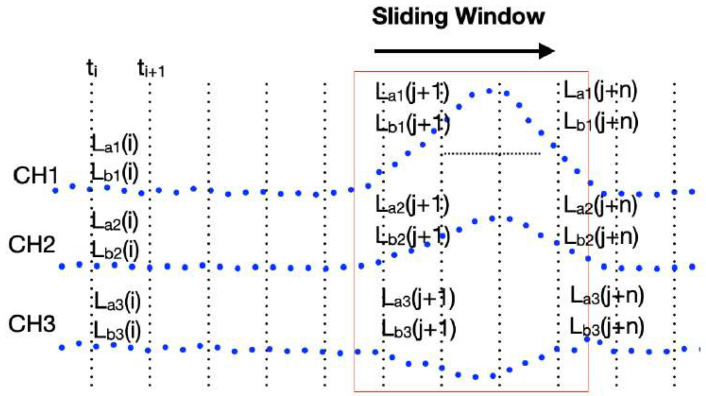
Schematic of the multi-channel synchronous extraction of vehicle-induced dynamic strains.

Figure 9 depicts a schematic diagram of the vehicle-induced dynamic strains captured by multi-channel synchronous acquisition. Figure 9 can also be used to illustrate the algorithm of edge computing. CH1 serves as the primary strain-monitoring channel, while CH2 and CH3 serve as secondary strain-monitoring channels. Due to limitations in the MCU memory during firmware development, not all the data are stored. Instead, the time axis is divided into fixed intervals, such as 1 s, and then during each sampling, the relative minimum and maximum values of CH1 within that 1 s interval and their corresponding occurrence times are recorded. This is referred to as the critical data of the interval. In Figure 9, from the time point ti to ti+1, there will be 100 points for the case with a sampling frequency of 100 Hz, and only the maximum and the minimum strains within the interval are stored in L_a1_(i+1) and L_b1_(i+1), respectively. Then, at the time points when the relative maximum and minimum strains occur in CH1 within the interval, CH2 and CH3 will simultaneously record the strain values at that time in L_a2_(i+1), L_a3_(i+1), L_b2_(i+1), and L_b3_(i+1), respectively. The same procedure is applied to search for and record the relative maximum and minimum values of different intervals using a sliding window approach. By dynamically storing the critical data of n intervals, where n intervals should cover the strain response induced by vehicles passing over the bridge. It is worth emphasizing that this “n” value can be adjusted remotely as needed.

According to the aforementioned algorithm, the firmware only needs to interpret whether the disturbance exceeds the threshold from the latest n intervals of key data in each window movement (every 1 s). If it exceeds the threshold, it is termed a disturbance event, and the maximum strain within the latest n intervals containing this disturbance event is obtained. From the above recording method, it is known that the occurrence time point of the maximum strain in CH1 coincides with the time points recorded by CH2 and CH3. The firmware then calculates the average value of the key data in each interval of CH1 that is unaffected by disturbances, and it uses it as a reference strain value for calculating the strain variation for each channel. Thus, subtracting the reference strain value of the unaffected intervals from the maximum strain caused by disturbances yields the maximum strain variation for each channel during that disturbance event. Within each minute, the main channel (CH1) will compare disturbance events to find the one with the greatest strain variation, along with the strain variation of the secondary channels at the corresponding time points. This information is then transmitted to the cloud as key data for that minute.

**Figure 10:**
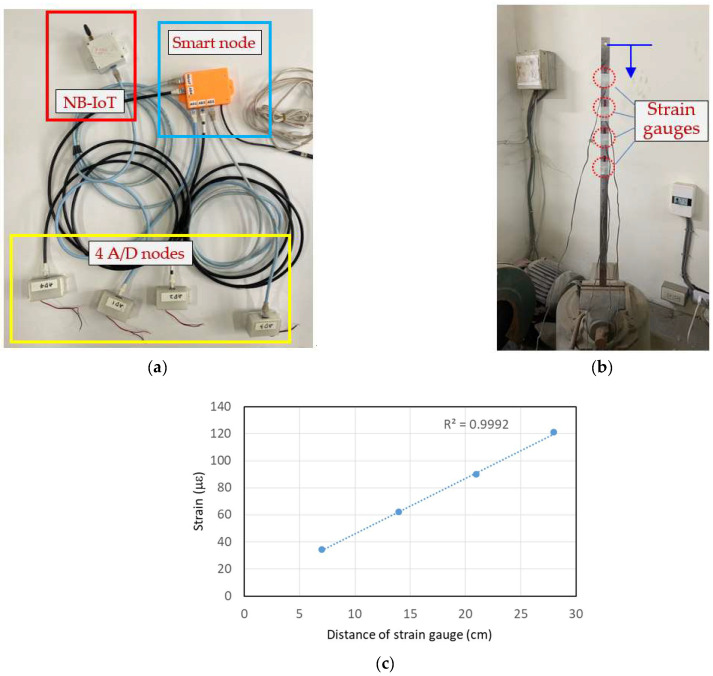
Multi-channel dynamic strain gauges’ edge-computing synchronized testing: (**a**) 4-channel data acquisition module; (**b**) testing apparatus; and (**c**) test results.

To validate the synchronous functionality of the aforementioned multi-channel dynamic strain gauges with edge computing, the four-channel dynamic strain data acquisition module depicted in Figure 10a was installed on a cantilever beam with four resistance strain gauges, as shown in Figure 10b. Before the experiment, the distances between each strain gauge and the point of force application (marked by the blue lines in Figure 10b) were recorded as 7, 14, 21, and 28 cm, respectively. A push-and-return disturbance lasting approximately 3 s was applied at the point of force application. The experimental results showed individual strains corresponding to the 4 strain gauges of 34.31, 62.07, 89.79, and 120.84 με. By setting the distances of the strain gauges and their corresponding measured strains as the horizontal and vertical coordinates, respectively, the relationship curve shown in Figure 10c can be plotted. As expected, the strain response is linearly related to the distance, indicating that the dynamic strains output by the four channels are indeed synchronous.

## 4. On-Site Application of Multi-Channel Synchronized Dynamic Strain Gauges

### Description of the Prestressed Box-Girder Bridge

Considering the significant prestress loss expected to occur during the first year after prestress application due to concrete shrinkage and creep, the on-site experiment was carried out on a newly completed box girder. This aims to observe whether the N.A. position changes due to prestress loss. By installing dynamic strain gauges at two positions on the top and bottom of the girder section and recording the strain responses caused by passing vehicles, information about the N.A. position can be obtained. Over time, one can observe whether the N.A. position moves upward due to prestress loss.

**Figure 11:**
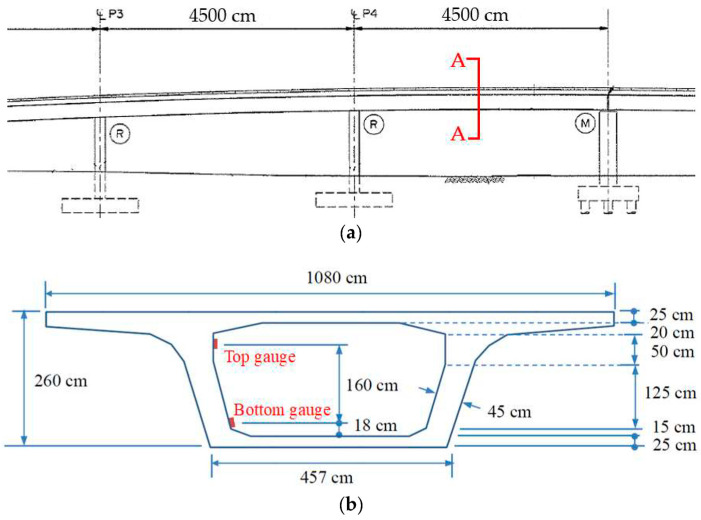
The monitoring target girder: (**a**) elevation view; and (**b**) cross-sectional dimensions.

**Figure 12:**
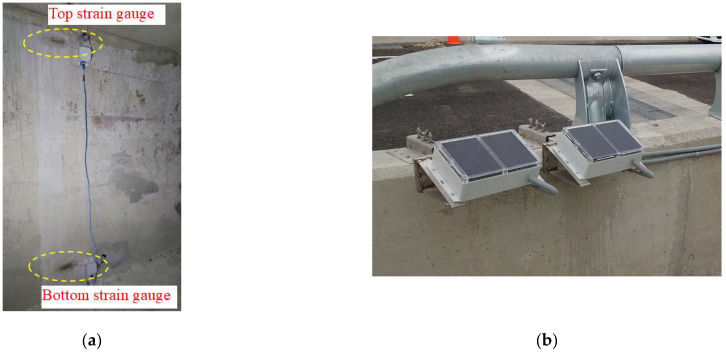
Photos of the on-site instrument installation: (**a**) dynamic strain gauges; and (**b**) solar power module.

This field study selected a box girder at Interchange Ramp 2 of Taiwan Freeway No. 4 as the monitoring target. The application of prestress to the bridge girder was conducted on 1 September 2021. Figure 11a,b, respectively, show the elevation and cross-section of the monitored bridge girder. The dynamic strain gauges were installed at the midpoint of the girder, as indicated in Figure 11a at section A-A. Figure 11b illustrates the dimensions of the cross-section and the installation height of the dynamic strain gauges. Based on the cross-sectional dimensions, the theoretical position of the N.A. can be calculated as 170.5 cm from the bottom of the girder. Figure 12a,b depict the installation photos of the dynamic strain gauges inside the box girder on-site, along with the solar power supply module.

**Figure 13:**
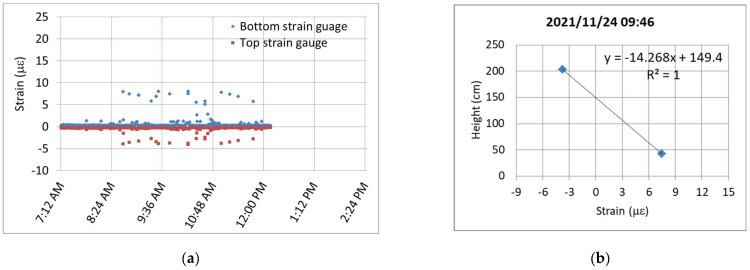

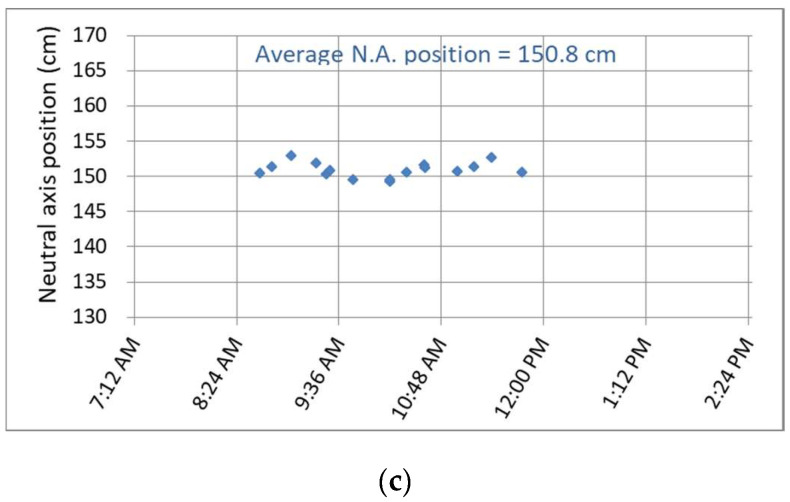
Dynamic strain response caused by the traffic of construction vehicles on 24 November 2021: (**a**) data measured by the upper and lower strain gauges; (**b**) calculation of the N.A. position; and (**c**) N.A. position distribution.

After the completion of the installation of the dynamic strain gauges on 24 November 2021, the dynamic strain responses caused by passing construction vehicles on the bridge were observed, as shown in Figure 13a. The figure illustrates the tensile strains (blue dots) and compressive strains (red dots) at the bottom and top strain gauges, respectively, when subjected to the loading of vehicles. The N.A. position can be determined either by calculation using Equation (1) or by the intercept of the linear regression in Figure 13b. Figure 13c depicts the distribution of the N.A. positions calculated from only the strains at the bottom of the box girder that exceeded 5 με, induced by heavy construction vehicles. The average position of the N.A. is approximately 150.8 cm. This average value can serve as a reference for subsequent monitoring.

**Figure 14:**
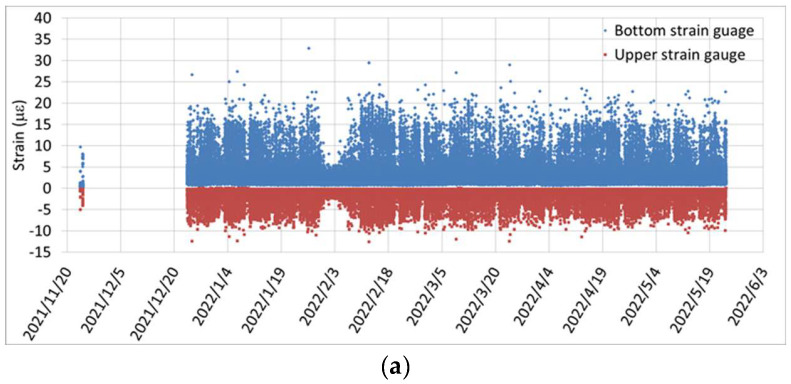

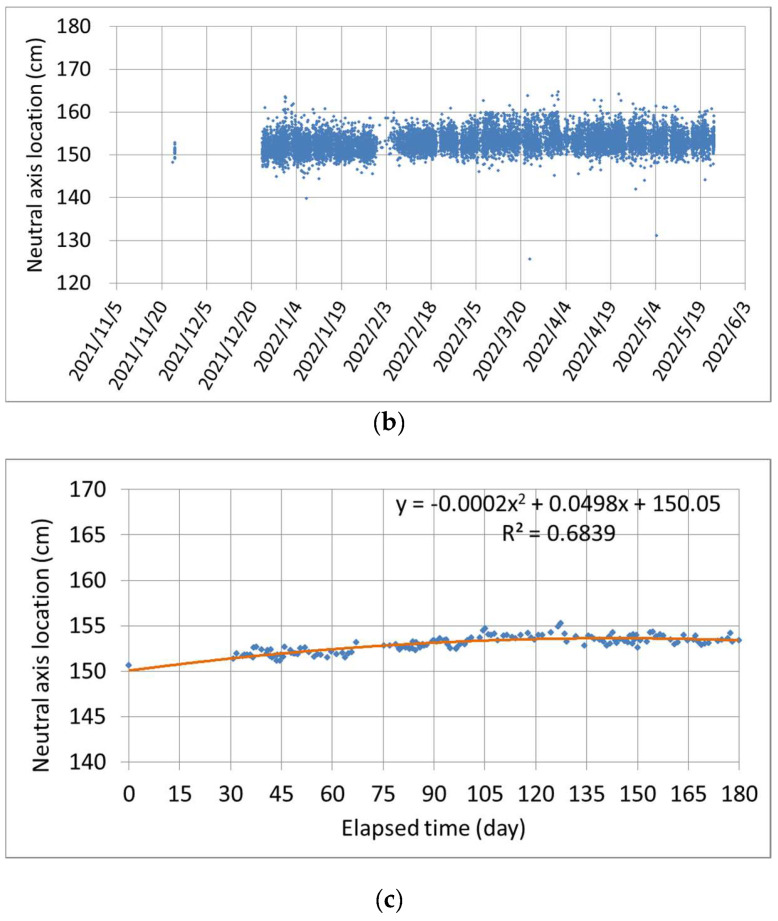
Continuous monitoring of the dual-channel synchronized dynamic strain responses: (**a**) measurement data from the upper and lower strain gauges; (**b**) distribution of N.A. positions; and (**c**) trend of the N.A. position over time.

Figure 14a illustrates the monitoring data collected continuously for six months after the commencement of traffic. The figure clearly depicts the strain responses (blue dots for the bottom strain gauge and red dots for the top strain gauge) caused by passing vehicles. Figure 14b displays the variation in the N.A. position over time, calculated from the measured strain responses of the upper and lower strain gauges. In Figure 14b, the N.A. position fluctuates within a certain range over time. This phenomenon is attributed to the temperature gradient changes within the box girder [20,21].

It is worth noting that, based on the on-site monitoring data shown in Figure 14b, the N.A. position fluctuates between 145 and 160 cm from the bottom of the girder. These values are consistently lower than the theoretical N.A. position of 170.5 cm, indicating a downward shift in the N.A. position due to the prestressing effect of the box girder. Additionally, Figure 14b demonstrates a gradual upward trend in the N.A. position over time, starting from 150.8 cm before the bridge was opened to traffic. To illustrate this trend more clearly, a simplified plot of the N.A. trend was created by taking a moving average of 100 data points and plotting it every 100 data points, as shown in Figure 14c. From Figure 14c, it is evident that the N.A. position is indeed slowly moving upwards. This phenomenon confirms that shortly after the completion of the prestressed girder, there is a sustained slow loss of prestress due to significant concrete shrinkage and creep effects. This result aligns with that obtained from the laboratory experiment, which indicates that the N.A. position tends to move upward as the prestress decreases.

**Figure 15:**
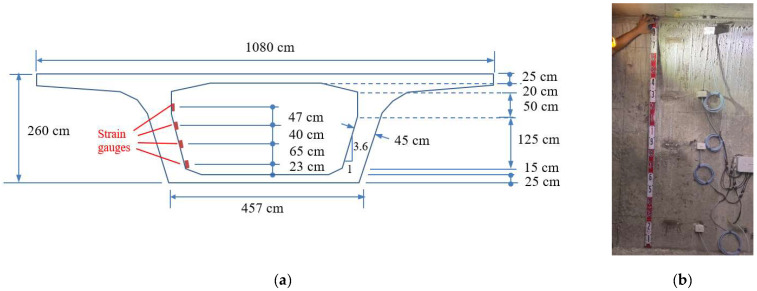
Four-channel synchronized dynamic strain gauges: (**a**) configuration heights of the dynamic strain gauges; and (**b**) photo of the completed instrument installation.

**Figure 16:**
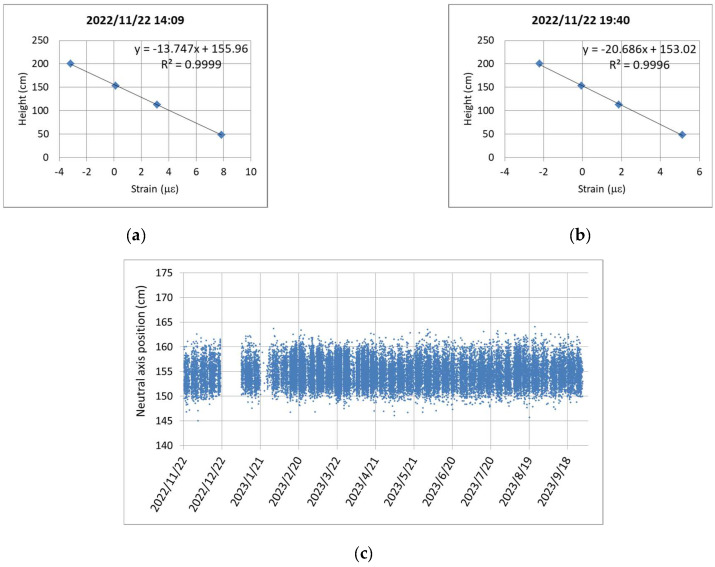

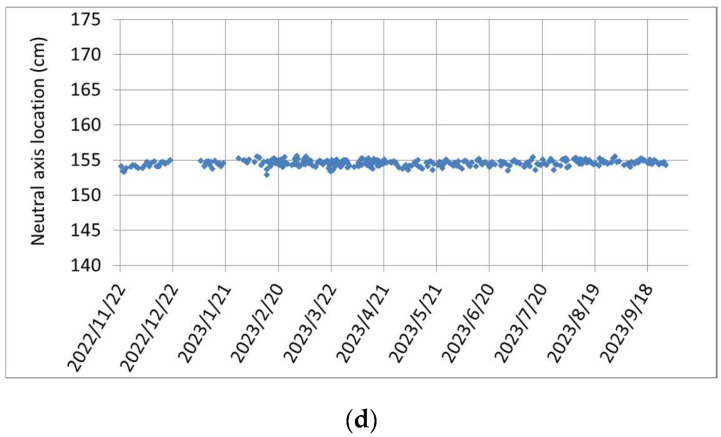
Continuous monitoring of the four-channel synchronized dynamic strain responses: (**a**) linear regression of the four points at 14:09; (**b**) linear regression of the four points at 19:40 (**c**) distribution of the N.A. positions; and (**d**) simplified trend of the N.A. positions.

The monitoring device for the box girder was upgraded to the newly developed four-channel synchronized dynamic strain gauges in November 2022. Figure 15a,b, respectively, depict the schematic diagram and completion photo of the installation heights of the four-channel dynamic strain gauges. For the strain gauges attached on an inclined plane, the inclined lengths must be converted into vertical heights for calculating the neutral axis position. From Figure 15a, it can be inferred that the heights of the four strain gauges from the bottom of the beam are 48, 113, 153, and 200 cm, respectively. Figure 16a,b show the linear regression results of the four-channel strain responses measured at two specific time points and their corresponding installation heights. According to the cross-sectional height at zero strain, the intercepts of the linear regression equations in Figure 16a,b, which are 155.96 and 153.02 cm, respectively, denote the N.A. positions measured at those two time points.

In order to reduce the influence of the measurement signal noise on the calculation of the N.A. position, it is set that the dynamic strain response at the bottom must be greater than 5 με, and the coefficient of determination (R^2^) of the linear regression equation must be greater than 0.995 to be included in the data used for calculating the N.A. position. It is believed that the N.A. positions obtained from the four-channel strain gauge data are more reliable than those obtained from the two-channel strain gauge data. Figure 16c shows the monitoring results of the N.A. position from 22 November 2022 to 27 September 2023. In Figure 16c, it can be observed that the measured N.A. position fluctuates between approximately 150 and 160 cm. Apart from a slight upward movement in the first month after the completion of the instrument installation, the N.A. position subsequently demonstrates a considerable stability, indicating that prestressed beams completed for over a year are less susceptible to prestress loss caused by concrete shrinkage and creep. To simplify the data in Figure 16c, a continuous moving average of 100 data points was used, and one data point was selected every 100 data points to redraw the trend of the N.A. position, as shown in Figure 16d. Similarly, it can be observed that the N.A. position slightly increases in the first month, but afterwards, it stabilizes at around approximately 155 cm, remaining below the theoretical N.A. position of 170.5 cm.

## 5. Conclusions

In this study, the N.A. position was investigated as an indicator for prestress variation monitoring. Laboratory experiments were conducted to investigate the correlation between the prestress magnitude and the N.A. position. A newly completed box girder was chosen as the subject for on-site measurement of the N.A. position using multi-channel synchronized dynamic strain gauges shortly after the prestress was applied. To enhance the efficiency of the field monitoring system, edge-computing technology was utilized to develop multi-channel synchronized dynamic strain gauges.

The following conclusions are drawn from the results of this study:

1. The results of the laboratory test showed that the N.A. position tends to decrease with an increasing prestress force. In other words, if prestress loss occurs in the beam, an upward movement of the N.A. position should be observed.
2. The developed multi-channel synchronized dynamic strain gauges with edge computing can transmit only the key data every minute. These key data represent the maximum dynamic strain induced by the heaviest vehicle within that minute. This approach significantly enhances the monitoring efficiency and stability, allowing for the replacement of traditional computer-based monitoring systems with microcontroller systems in the field.
3. Continuous monitoring data concerning the dual-channel synchronized dynamic strains depicts the tensile strains and compressive strains at the bottom and top strain gauges, respectively, when subjected to the loading of vehicles. The N.A. position can be obtained from the measured strain responses of the top and bottom strain gauges. The N.A. position fluctuates within a certain range over time. This phenomenon is attributed to the temperature gradient changes within the box girder.
4. Based on the on-site monitoring data, the measured N.A. position of the box girder is lower than the theoretical N.A. position, indicating a downward shift in the N.A. position due to the prestressing effect. Additionally, it is observed that the N.A. position is slowly moving upwards. This phenomenon confirms that shortly after the completion of the girder, there is a slow sustained loss of prestress due to significant concrete shrinkage and creep effects. This result aligns with that obtained from the laboratory experiment, which indicates that the N.A. position tends to move upward as the prestress decreases.
5. This study utilized a newly developed four-channel synchronized dynamic strain gauges for on-site applications. The coefficient of determination (R-squared) for the linear regression equations of the four-channel strain data must be greater than 0.995 to be included in the calculation of the N.A. position. It is believed that the N.A. positions obtained from the four-channel strain gauge data are more reliable than those obtained from the two-channel strain gauge data.

## Figures and Tables

**Table 1:** Strain responses of the top and bottom steel bars caused by the load test and the calculated N.A. position.

	ε_t_ (με)	ε_b_ (με)	N.A. Position (cm)
Loading 500 kgf	−12.32	23.16	28.37
Loading 500 kgf	−11.89	24.34	29.04
Unloading 500 kgf	12.60	−22.92	28.11
Unloading 500 kgf	13.52	−24.35	28.03
Average			28.39

**Table 2:** Experimental results of the T-beam under different prestress conditions.

Prestress Force (tf)	Loading Case	ε_t_ (με)	ε_b_ (με)	N.A. Position (cm)
8.13	Loading 500 kgf	−13.15	27.11	29.07
	Unloading 500 kgf	12.59	−24.33	28.58
12.23	Loading 500 kgf	−12.84	25.11	28.67
	Unloading 500 kgf	12.75	−23.79	28.31
23.87	Loading 500 kgf	−11.53	20.49	27.93
	Unloading 500 kgf	11.55	−20.51	27.92
27.57	Loading 500 kgf	−11.39	21.73	28.48
	Unloading 500 kgf	11.19	−20.10	28.01
33.33	Loading 500 kgf	−11.06	18.64	27.49
	Unloading 500 kgf	10.77	−18.44	27.62
44.52	Loading 500 kgf	−10.09	16.58	27.29
	Unloading 500 kgf	10.16	−16.22	27.06

## Data Availability

Some or all the data, models, or code that support the findings of this study are available from the corresponding author upon reasonable request.
